# Social Organization and Co-operation as a Means of Advancing Medical Science

**Published:** 1875-01-15

**Authors:** 


### SOCIAL ORGANIZATION AND CO-OPERATION AS A MEANS OF ADVANCING MEDICAL SCIENCE.

THERE are probably but few reading and thinking men in the profession who have not been impressed with the fact, that through all the past, much time and labor have been substantially lost or wasted either in repeating experiments and observations already fully made by others, or in prosecuting inquiries so imperfectly planned and partially executed, as to render the results of very little value.

Our literature is full of facts, observations, and the results of experiments relating to almost every department of medical science, which, from their isolated character or incompleteness, are quite as likely to be made the basis of erroneous conclusions as otherwise.

Very much of this might be obviated, and the scientific interests of the profession much more rapidly advanced, if our social organizations could be rendered more complete, and more intimately united with each other. Many of the most important questions in the department of etiology and public hygiene, can only be answered reliably by a careful comparison of facts observed at many different localities simultaneously, and in accordance with a uniform plan. If members of the profession could be more generally induced to become members of the local, state, and national associations, and all these more perfectly brought into co-operation by uniform representation from the more local in the more general organizations, it would enable measures and plans of investigation of the highest importance to be adopted and carried out on such a scale of magnitude and uniformity, as to ensure the most reliable results.

The present national, state, and local medical societies, already present the form of organization needed; but the state and local societies need multiplying and greatly increasing their membership, thereby including and interesting a larger proportion of the active practitioners in all parts of the country.

This work is steadily progressing—and at the last meeting of the American Medical Association, the work of planning special courses of investigation was fairly begun, both in the section on practical medicine and materia medica, and in that on public hygiene and state medicine. We hope to see this followed closely at the next meeting, and every successive year, until at each meeting of all our state societies, as well as of the American Medical Association, the time shall be mainly occupied in receiving, digesting, and recording the results of well planned original or primary investigations; and the adoption of new plans for the future, instead of listening to long papers or reports compiled mostly from the current periodical literature. One of the results of a movement made in the section on practical medicine at Detroit is, a proposition before Congress to add to the records of the Signal Service Bureau, the electrical and ozonic conditions of the atmosphere, and to permit the records of that bureau to be used in aid of sanitary science. If the proposition receives the sanction of Congress, it will open the way for a most important advance in our knowledge of the causes of all zymotic diseases.

